# In vitro models of multiple system atrophy from primary cells to induced pluripotent stem cells

**DOI:** 10.1111/jcmm.13563

**Published:** 2018-03-04

**Authors:** Elena Abati, Alessio Di Fonzo, Stefania Corti

**Affiliations:** ^1^ Department of Pathophysiology and Transplantation (DEPT) Dino Ferrari Centre Neuroscience Section Neurology Unit IRCCS Foundation Ca' Granda Ospedale Maggiore Policlinico University of Milan Milan Italy

**Keywords:** in vitro models, induced pluripotent stem cells, multiple system atrophy, neurodegeneration, oligodendrocytes

## Abstract

Multiple system atrophy (MSA) is a rare neurodegenerative disease with a fatal outcome. Nowadays, only symptomatic treatment is available for MSA patients. The hallmarks of the disease are glial cytoplasmic inclusions (GCIs), proteinaceous aggregates mainly composed of alpha‐synuclein, which accumulate in oligodendrocytes. However, despite the extensive research efforts, little is known about the pathogenesis of MSA. Early myelin dysfunction and alpha‐synuclein deposition are thought to play a major role, but the origin of the aggregates and the causes of misfolding are obscure. One of the reasons for this is the lack of a reliable model of the disease. Recently, the development of induced pluripotent stem cell (iPSC) technology opened up the possibility of elucidating disease mechanisms in neurodegenerative diseases including MSA. Patient specific iPSC can be differentiated in glia and neurons, the cells involved in MSA, providing a useful human disease model. Here, we firstly review the progress made in MSA modelling with primary cell cultures. Subsequently, we focus on the first iPSC‐based model of MSA, which showed that alpha‐synuclein is expressed in oligodendrocyte progenitors, whereas its production decreases in mature oligodendrocytes. We then highlight the opportunities offered by iPSC in studying disease mechanisms and providing innovative models for testing therapeutic strategies, and we discuss the challenges connected with this technique.

## 1 INTRODUCTION

Multiple‐system atrophy, also known as MSA, is an adult onset severe neurodegenerative disease characterized by glial cytoplasmic inclusions (GCIs) and progressive cellular death in selected areas of central nervous system (CNS), more specifically the striatonigral, olivopontocerebellar and central autonomic pathways. The clinical presentation mirrors these alterations and comprises parkinsonism, cerebellar ataxia, pyramidal features and autonomic symptoms in various degrees.^1^ Two main clinical subtypes can be identified and characterized by either a prevalence of parkinsonian symptoms (MSA‐P) or a prevalence of cerebellar ataxia (MSA‐C).^2^

The estimated incidence ranges from 0.1 to 2.4 cases per 100 000 person‐years, the mean value being 0.6‐0.7/100 000.^1^, ^3^ Prevalence has been reported to span between 1.9 and 4.9 per 100 000, according to different population studies.^4^, ^5^ MSA‐P accounts for approximately two‐thirds of the cases in European countries, with regional differences,^2^, ^6^, ^7^ whereas MSA‐C is far more common in Japan.^8^

Symptom onset occurs usually in the sixth decade, and the median survival from that time is estimated to be 9.8 years.^9^, ^10^

In the majority of cases, MSA appears sporadically in the population. However, some cases have been reported of Japanese, German and American families showing a genetic transmission of the disease.^11^, ^12^, ^13^, ^14^, ^15^, ^16^ Despite these findings, no disease‐causing mutation could be identified in these studies.

COQ2 is a gene that encodes the enzyme parahydroxybenzoate‐polyprenyl transferase, which catalyses one of the final reactions in the biosynthesis of coenzyme Q10 (ubiquinone). Intriguingly, whole genome sequencing of a Japanese family and large case‐control series revealed that COQ2 variants may be associated with an increased risk of MSA in East Asia.^17^, ^18^

However, other groups failed to report the same correlation between MSA and COQ2 variants in western countries.^19^, ^20^, ^21^ These findings notwithstanding, the role of COQ2 in the pathogenesis of MSA remains unclear.

In addition to that, Gaucher disease‐causing mutations of GBA (glucocerebrosidase) gene were recently sequenced in 969 MSA patients and in 1509 control subjects, demonstrating an association between many GBA variant and MSA, as it happens in Parkinson's disease (PD).^22^

Finally, genomewide association studies (GWAS) have pointed out possible correlation between alpha‐synuclein encoding gene (SNCA) polymorphisms and MSA.^23^, ^24^ However, the largest MSA GWAS did not find any relevant association.^25^ Additional studies with more samples may shed new light on potentially significant associations in future.

At a histopathological level, the main features of MSA are selective neuronal loss and axonal degeneration, alpha‐synuclein immunoreactive inclusions and gliosis. The pathologic hallmark of MSA is insoluble GCIs, whose main proteinaceous component is alpha‐synuclein.^26^ Thus, the presence of GCIs characterizes MSA as a “synucleinopathy,” together with PD and Lewy Body Dementia (LBD). In contrast to inclusions in PD and LBD, however, GCIs predominantly accumulate in oligodendrocytes.^27^ Other relevant proteins that can be found in aggregates are p25alpha/TPPP, LRRK2 and tau protein.^28^, ^29^

GCIs are diffusely distributed in specific anatomical regions of the CNS. More specifically, the pyramidal, striatonigral, corticocerebellar and preganglionic autonomic systems are affected in a preferential manner.^30^ GCIs are also widely present in the motor cortex, despite mild levels of cellular degeneration.^31^

Nonetheless, glial alterations in the white matter are not limited to oligodendrocytes, but involve also astrocytes and microglia. In fact, the extent of reactive astrocytosis and activated microglia parallels the degree and anatomical distribution of GCIs and neurodegeneration.^32^, ^33^

Neuronal cytoplasmic inclusions (NCIs) and neuronal nuclear inclusions (NNIs) can also be found in MSA, although less frequently than GCIs.^34^ They are mainly found in cortical, subcortical, cerebellar and brainstem nuclei, being especially prevalent in the pons and inferior olivar nucleus.^35^

## 2 THE PATHOGENESIS OF MULTIPLE SYSTEM ATROPHY

**Figure 1:**
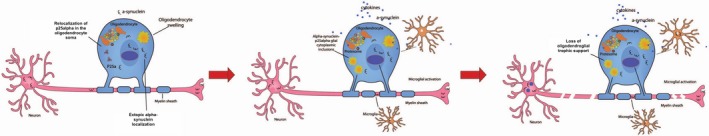
Hypothetical features of multiple system atrophy pathophysiology. Early in the course of the disease, p25alpha relocalizes into the oligodendroglial soma. Subsequently, altered expression or uptake of alpha‐synuclein in oligodendrocytes and interaction with p25alpha causes the formation of glial cytoplasmic inclusions, which eventually determine oligodendroglial dysfunction and loss of neurotrophic support. Misfolded alpha‐synuclein can also be taken up by neurons, with the formation of neuronal cytoplasmic and nuclear inclusions. Defective autophagic clearance mechanisms promote the accumulation of intracellular alpha‐synuclein at an increased rate. Together with microglial activation, these factors ultimately lead to neurodegeneration and neuronal death

To this day, the pathogenic mechanisms that lead to the development of MSA are yet to be unravelled. Nevertheless, there is compelling evidence that MSA is a primary oligodendrogliopathy, which encompasses alpha‐synuclein misfolding and aggregation, early myelin dysfunction and axonal disease (Figure 1).^36^, ^37^

Early myelin dysfunction is suggested by the finding that p25alpha (TPPP), usually found in myelin sheaths, relocates in the oligodendrocyte soma in the first stages of the disease.^38^ Moreover, the co‐localization of p25alpha and MBP is noticeably decreased in MSA brains, and total MBP content is reduced. The presence of p25alpha in the body of the cell could then enhance the aggregation of alpha‐synuclein.^39^

The interaction between p25alpha and alpha‐synuclein may result in the formation of GCIs, which in turn interferes with oligodendrocyte survival and neuronal support. Alpha‐synuclein is thought to play a major pathogenic role in the disease, although it is unclear whether it is normally expressed in oligodendrocytes. Analysis of postmortem healthy controls' brains yielded contrasting results: in situ hybridization techniques failed to detect alpha‐synuclein mRNA expression in glial cells,^40^, ^41^ whereas a recent study identified SNCA mRNA in oligodendrocytes using qPCR.^42^ Djelloul and colleagues sought evidence of the expression of alpha‐synuclein in oligodendrocyte lineages derived from mouse embryonic stem cells (ESC) and human‐induced pluripotent stem cells (iPSCs) and in oligodendrocytes from mice postnatal forebrains. They found that alpha‐synuclein was expressed in oligodendrocytes progenitors, but its levels decreased during maturation and were absent in the final stages of differentiation.^43^

The origin of GCIs' alpha‐synuclein in MSA therefore is not clear. One hypothesis is that it results from endogenous overexpression in oligodendrocytes, notwithstanding that many studies contradict this view. The in situ hybridization studies previously described did not retrieve SNCA mRNA in MSA patients' white matter.^40^, ^41^ Analysis by qPCR revealed the presence of alpha‐synuclein transcripts but the difference with PD patients and healthy controls was not statistically significant.^42^ Furthermore, in vitro culturing of MSA‐derived iPSCs showed that, as in control and PD‐derived lines, alpha‐synuclein was expressed in the first stages of oligodendrocytes' development but not in the premyelinating phase.^43^ On the contrary, upregulation of synuclein expression has been demonstrated to impair the correct maturation of the oligodendrocyte.^44^

Another theory claims that alpha‐synuclein might be produced in neurons and then taken up by oligodendrocytes. Several studies advocate this hypothesis, showing that oligodendrocytes are capable to absorb neuronal secreted or exogenously added alpha‐synuclein both in vitro and in vivo.^45^, ^46^ Under physiological conditions, alpha synuclein is primarily produced by neuronal cells as an unfolded protein.^47^ Several studies showed that alpha‐synuclein aggregates can transmit from neuron to neuron,^48^ astroglial cells 49 and oligodendroglial cells,^47^ thus supporting the hypothesis of neuronal transmission. Oligodendrocytes are then able to take up alpha‐synuclein via endocytotic mechanisms.^50^ Hansen and colleagues provided evidence that alpha‐synuclein may propagate from cell to cell and exert a seeding effect on the endogenous protein, thereby contributing to the spread of the pathology.^51^ This discovery, along with a recent study by Prusiner and colleagues, suggests that alpha‐synuclein could disseminate in a prion‐like fashion.^52^

The connection between alpha‐synuclein accumulation and neurodegeneration is still a matter of debate. According to some studies, alpha‐synuclein may have a role in activating intrinsic and extrinsic apoptotic pathways within oligodendrocytes.^53^, ^54^ Oligodendrocytes' dysfunction then affects neuronal survival,^55^ for example through a reduction in glial derived neurotrophic factor (GDNF).^56^ Moreover, in vitro studies demonstrated that alpha‐synuclein aggregates directly induce neuronal dysfunction and apoptosis.^48^, ^57^, ^58^ Desplats and colleagues demonstrated the formation of inclusion bodies in neurons after alpha‐synuclein uptake, possibly through lysosomal dysfunction, and apoptosis of involved neurons.^48^ Another study showed that exogenous alpha‐synuclein fibrils induce pathological alpha‐synuclein accumulation, neuron loss and diminished levels of synaptic proteins.^58^ Klucken and colleagues found that inhibition of autophagy with bafilomicin A1 increased toxicity, measured as the release of adenylate kinase, in neurons transfected with C‐terminal modified α‐synuclein.^59^ Impairment of autophagic pathways has already been reported in nigral neurons of PD patients' brains 60 and evidence for a potential role of autophagy in MSA pathogenesis is emerging from in vitro and in vivo studies.^61^, ^62^

Furthermore, there is evidence that also inflammatory response, whose main actors are microglia and astrocytes, plays an active role in perpetuating and extending brain damage. Activated Iba‐1‐positive microglia and GFAP‐positive astrocytes are shown to colocalize with GCIs.^63^ Moreover, treatment of primary astrocytes with alpha‐synuclein triggered astrogliotic changes, whereas extracellular alpha‐synuclein is phagocytosed by microglia inducing microgliosis and production of reactive oxygen species (ROS).^64^ In particular, the Toll‐like receptors 2 and 4 are reported to interact with alpha‐synuclein and exhibit upregulation in MSA patients.^65^, ^66^, ^67^ Finally, it is suggested that the release of cytotoxic products by activated glia may favour alpha‐synuclein misfolding and aggregation.^68^

In the light of the recent progress on the pathophysiological mechanism, we now have a better understanding of how oligodendrocytes' dysfunction and alpha‐synuclein accumulation develop in the human CNS. However, as symptoms in MSA patients appear to be caused by neuronal degeneration, and not by demyelination in oligodendrocytes, the molecular interactions between the degenerated oligodendrocytes have to be better elucidated.

Among the hypothesized mechanisms, oligodendrocytes' dysfunction might cause neuronal death through the activation of neuroinflammatory mechanisms 63, 64, 65, 66, 67 and the loss of neurotrophic support.^56^ Neuronal dysfunction because of α‐synuclein inclusions^48^, ^57^, ^58^ and autophagy impairment^60^, ^61^, ^62^ also act synergistically, leading to neuronal death in the striatonigral, olivopontocerebellar and central autonomic pathways.^31^ This secondary neurodegeneration may explain the typical symptoms observed in MSA patients, the lack of response to L‐DOPA and the rapid progression of this disease.

Demyelination plays an important role in advanced MSA,^33^ and recent studies found that intracellular alpha‐synuclein delays oligodendrocytes maturation and myelination by downregulating myelin‐gene regulatory factor and myelin basic protein (MBP).^44^, ^69^ Myelin dysregulation is often followed by axonal degeneration,^36^ as demonstrated by transgenic animal models.^70^, ^71^

Several other mechanisms have been examined as potentially pathogenic, such as proteasome system inhibition.^72^ A recent field of investigation is focusing on exosomes, small extracellular vesicles which are involved in the reciprocal communication between oligodendrocytes and neurons, in neural trophic support and in the regulation of microglial response. Exosomes appear able to catalyse and accelerate the nucleation of alpha‐synuclein. Exosomes are also suspected of playing a role in the prion‐like spread of proteins, such as alpha‐synuclein, in neurodegenerative diseases.^73^, ^74^, ^75^

In conclusion, many pathways, from gene expression to protein transport and inflammatory response, appear to be involved and to interact over the course of the disease. However, further investigations are needed to establish their precise role and weight in the pathogenesis of MSA.

## 3 INDUCED PLURIPOTENT STEM CELLS IN NEURODEGENERATIVE DISEASES

In 2006, Takahashi and Yamanaka for the first time successfully reprogrammed somatic cells, mouse fibroblasts at the beginning, into embryonic like cells, the so‐called iPSCs.^76^ The induction of pluripotency in vitro can be achieved by overexpression of defined cocktail of pluripotency‐associated genes, or reprogramming factors, namely Oct3/4, Sox2, Klf4 and c‐Myc (OSKM), that are transduced in somatic cells, usually fibroblasts, with a retroviral or lentiviral system.^77^, ^78^ This procedure allows to obtain cells similar to ESCs in morphology, proliferation, surface antigens, gene expression, epigenetic status and telomerase activity.

The mechanisms by which reprogramming factors exert their action have not been completely elucidated. Oct3/4 and Sox2 upregulate stemness genes and suppress differentiation‐associated genes, acting synergistically.^79^ The role of Klf4 and c‐Myc is less clear, but it has been proposed that Klf4 favours epithelial transition by binding to specific genes, while c‐Myc seems to be involved in the regulation of cellular proliferation, metabolism, and biosynthetic pathways.^80^, ^81^

The advantages of iPSCs over ESCs are numerous and relevant. Firstly, while the use of ESCs is limited by ethical concerns because of their embryonal origin, iPSCs are produced from adult cells and thus they overcome this issue. Secondly, they retain the same genetic makeup as the original source. Therefore, they allow the production of patient‐specific disease models and provide hope for the development of therapies based on autologous iPSCs transplantations.

The challenges of this technique are the relative low efficiency of the protocol, the risk of mutations following the genomic integration of transcription factors, the potential tumour risk if used as cell source for transplantation, and the possibility of incomplete reprogramming.

Remarkably, the discovery of iPSCs has paved the way for direct pharmacological reprogramming, a technique that was pioneered in 2016 by Zhang et al^82^ Further studies are needed to perfect this method. However, there is a hope that the absence of genetic manipulation could reduce the risk for genomic alterations.

iPSCs have already provided successful and patient‐specific models of different neurodegenerative diseases. Thanks to their pluripotent phenotype, iPSCs can be differentiated in the various cellular lines that are preferentially affected in the different diseases. Neuronal and glial models of PD,^83^, ^84^, ^85^, ^86^ Huntington's disease,^87^, ^88^, ^89^ amyotrophic lateral sclerosis^90^, ^91^, ^92^ and Alzheimer's disease^93^, ^94^, ^95^ have already been realized.

## 4 MODELLING MULTIPLE SYSTEM ATROPHY IN VITRO: PRIMARY CELL CULTURE AND LINES

One factor that makes MSA such a puzzling disease is the difficulty to obtain reliable disease models. This is mainly because of its complex neuropathologic features and to the lack of a recognized genetic background. Moreover, different cell lineages are involved. Despite the fact that aggregates are located mainly in oligodendrocytes, it is not clear whether alpha‐synuclein originates from neurons or glial cells. Both pharmacological (by stereotaxic injection of toxins) and transgenic mouse models have been developed in the past.^70^, ^71^, ^96^, ^97^, ^98^, ^99^, ^100^, ^101^, ^102^, ^103^, ^104^ However, those models were not able to precisely recapitulate biological and clinical features of the disease. Moreover, drugs that work in animals may not yield the same results in human trials. Thus, there is a strong need for a precise and human‐derived in vitro model of MSA.

### 4.1 Primary cell models

At first, in vitro studies were based on primary oligodendrocytic lineages, such as OLN‐93,^105^ derived from transformed rat oligodendrocytes or primary glial lineages, such as CG4,^61^, ^69^ from rat brain and U737,^106^ obtained from human glioblastoma‐astrocytoma specimens. Clonal cell lines were genetically modified so to overexpress alpha‐synuclein, as primary human and rat oligodendrocytes do not express significant levels of alpha‐synuclein (Table 1). In an early study, Stefanova and colleagues transfected glial cultures with vectors pDSred‐N1 or pEGFP‐N1 yielding plasmids encoding human wild‐type and C‐terminally truncated form of alpha‐synuclein. They found that the overexpression of human alpha‐synuclein produced widespread fibrillar α‐synuclein aggregates and cellular damage, and that C‐terminally truncated form is much more prone to form aggregates than the full‐length alpha‐synuclein. Unsurprisingly, they also observed that overexpression of alpha‐synuclein enhanced cell death rates and increased cell susceptibility to oxidative stress. Notably, these changes took place both in astrocytes and in oligodendrocytes.^106^

**Table 1:** In vitro models of multiple system atrophy

Authors	Starting cells	Main findings	Reference
Stefanova et al (2005)	U373 astrocytoma cell line and primary mixed rat glial culture overexpressing human WT α‐syn(1‐140) or C‐ terminally truncated α‐syn (1‐111) under the CMV promotor.	Presence of widespread fibrillar α‐syn aggregates, more numerous in cells expressing the C‐terminally truncated form; increased cell death rates; increased susceptibility to treatment with TNFα.	106
Kragh et al (2009)	Oligodendroglial cell line (OLN‐93) derived from primary Wistar rat brain glial cultures expressing human WT α‐syn or S129A or S129D mutant α‐syn with human WT p25α.	Coexpression of α‐syn and p25α causes microtubule relocalization to the perinuclear region; p25α‐mediated microtubule retraction requires low levels of α‐syn; α‐syn‐dependent microtubule retraction induces apoptotic markers with activation of caspase‐3 and nuclear chromatin condensation.	105
May et al (2014)	Primary rodent oligodendroglial cell line (CG4) expressing human WT α‐syn under the CMV promoter.	Intracellular α‐syn impairs OPC maturation in vitro; BDNF partially rescues OPC maturation.	69
Valera et al (2017)	Primary rodent oligodendroglial cell line (CG4) co‐infected with Lentivirus expressing human α‐syn or LV control and microRNA‐101 (miR‐101a‐3p) or control vector.	Autophagy inhibition in CG4 cells compared to controls; Lentiviral delivery of an antimiR‐101 construct reduces α‐syn‐induced autophagy deficits.	61
Djelloul et al (2015)	iPSCs obtained from donors' skin fibroblasts, differentiated into oligodendrocytes.	O4+, OLIG2+, PDGFRA+ oligodendrocyte progenitors express *Snca* and α‐syn; their expression decreases in MBP+, CNPASE+ premyelinating oligodendrocytes, without significant differences among control and patient lines.	43

Abbreviations: α‐syn, alpha‐synuclein; BDNF, brain‐derived neurotrophic factor; OPC, oligodendrocyte progenitor cell; WT, wild type.

Subsequently, Kragh and colleagues obtained a successful model of glial degeneration by co‐expressing human alpha‐synuclein and p25alpha in a rat oligodendroglial cell line, using the vector pET‐11d.^106^ As explained above, p25alpha appears to be implicated in early myelin dysfunction in MSA and to induce alpha‐synuclein aggregation.^39^ In their study, they observed that coexpression of alpha‐synuclein and p25alpha caused a retraction of microtubules from the cellular processes to the perinuclear region after the transfection. This phase was followed by a lasting induction of apoptotic markers with microscopically detectable caspase‐3 activation and nuclear chromatin condensation. Microtubules rearrangement was dependent on coexpression of alpha‐synuclein and p25alpha, as cells tolerated alpha‐synuclein expression and reacted only marginally to the expression of p25alpha. Moreover, it was observed that phosphorylation of alpha‐synuclein at Ser‐129 is critical for its toxic effect, as the expression of phosphorylation‐deficient S129A mutant resulted in absent microtubules retraction. This is an important finding as Ser‐129 phosphorylation hallmarks alpha‐synuclein pathology in tissues^107^ and was shown to mediate alpha‐synuclein inclusion formation in neurons.^108^

May and colleagues also developed a cellular model of MSA by overexpressing human wild‐type alpha‐synuclein in a rodent oligodendroglial cell line, GC4.^69^ The expression of alpha‐synuclein was shown to impair oligodendrocyte progenitor cells (OPCs) maturation, as human alpha‐synuclein‐expressing oligodendrocytes demonstrated abnormal branching, a lower number of MBP positive cells and a reduced intracellular MBP content at the final stages of differentiation compared to controls. These results suggest that accumulation of alpha‐synuclein in OPCs may result in downregulation of myelin‐associated genes.

The group also found out that OPC content is increased in the striatum of MSA‐P patients and that mice overexpressing human wild‐type alpha‐synuclein also display an increased number of striatal OPCs. Thus, they hypothesized that alpha‐synuclein may impair adult oligodendrogenesis, preventing OPCs from remyelination and contributing to MSA pathogenesis. Furthermore, the group found that BDNF mRNA is significantly reduced in the striatum of MBP transgenic mice, and that the supplementation of BDNF in vitro to transfected oligodendroglial cells partially rescues early OPCs' maturation, but lacks the potential to induce myelination.

Recently, Valera and colleagues generated a rodent oligodendroglial cell line, CG4, co‐infected with Lentivirus expressing human alpha‐synuclein or control and microRNA‐101 (miR‐101a‐3p) or control vector.^61^ The aim of the study was to investigate the potential pathogenic role of microRNA‐101 dysregulation in in vivo and in vitro models of MSA. Dysregulation of autophagy appears to be implicated in the pathogenesis and pathology of synucleinopathies,^60^, ^62^ and alterations in microRNA‐101 have been strongly associated with autophagy impairments in human cancer models.^109^, ^110^ They found that the coinfection of cells with alpha‐synuclein and miR‐101 led to autophagy inhibition (measured as a decrease in autophagy proteins Beclin 1 and LC3 levels and an increase in p62 protein levels) in CG4 cells compared to controls. To confirm these results, CG4 were then infected with a lentiviral construct expressing an antisense sequence against miR‐101, which resulted in a decrease in miR‐101. Co‐infection with alpha‐synuclein and antimiR‐101 induced an increase in the LC3 signal, and a significant decrease in the intracellular accumulation of alpha‐synuclein, suggesting that the repression of miR‐101 may effectively promote synuclein clearance in cellular models. The group also observed an increase in miR‐101 levels and a decrease in autophagy‐associated proteins in the striatum of MBP‐alpha‐synuclein transgenic mice. Interestingly, autophagy‐associated proteins co‐localized with alpha‐synuclein and the oligodendroglial marker Olig2. Stereotaxic injection of Lentivirus expressing anti‐miR‐101 resulted in an increase in the aforementioned autophagic proteins levels.

### 4.2 Achievements and limitations of primary cell models

These studies played a fundamental role in defining some key events in MSA pathogenesis. An important result that was observed is that overexpression of α‐synuclein in a human and rat primary mixed glial culture is sufficient to produce widespread fibrillar α‐synuclein aggregates and to trigger cellular stress and degeneration.^106^ In addition to that, it was demonstrated that the accumulation of alpha‐synuclein in OPCs may downregulate myelin‐associated genes and impair adult oligodendrogenesis.^69^ Moreover, it was noted that the co‐expression of alpha‐synuclein and p25alpha in OLN‐93 cells generated a successful model of MSA‐like degeneration, with alpha‐synuclein aggregation and microtubule retraction in association with activation of the apoptotic protein caspase 3.^105^ It was also demonstrated that microRNA‐101 overexpression induces autophagy impairment in alpha‐synuclein expressing cells and that its antagonization promotes synuclein clearance, thus paving the way for possible future therapeutic actions.^61^

However, studies based on primary cell cultures bear some limitations. Despite these models were able to replicate some pathological features of the disease, there is no evidence that alpha‐synuclein in MSA derives from endogenous overexpression in oligodendrocytes, and post‐mortem analyses of MSA patients' white matter by in situ hybridization and qPCR failed to retrieve a significant difference in the presence of SNCA mRNA compared to healthy controls.^40^, ^41^, ^42^ Moreover, while point mutations, duplications and triplications of SNCA locus are associated with both sporadic and familial variants of PD,^111^, ^112^, ^113^ no association was found between these mutations and MSA. GWASs pointed out a possible correlation between SNCA polymorphisms and MSA but subsequent studies yielded contrasting results.^23^, ^24^, ^25^ Thus, the validity of oligodendrocytic alpha‐synuclein upregulation as a model for the disease pathogenesis is limited.

In addition to that, common permanent oligodendrocyte cell lines have undergone various genetic modifications to drive immortal cell growth. For this reason, it may be difficult to determine whether results observed in tissue culture are artefacts or disease‐specific alterations.

## 5 MODELLING MULTIPLE SYSTEM ATROPHY IN VITRO BASED ON iPSC

Recently, iPSC obtained from patients have elicited new hopes in this field. Neurons and oligodendrocytes derived from MSA patients' iPSCs could yield new insights into the pathogenic process.

### 5.1 Differentiating iPSCs into oligodendrocytes

In this regard, one of the main challenges is represented by the nature of the cells targeted by MSA. In fact, oligodendrocytes' maturation in vitro is longer and more complex than neuronal one.^114^, ^115^ This is because neural stem cells (NSCs), in the absence of exogenous morphogens, commit to a dorsal telencephalic fate, more specifically to glutamatergic lineage.^116^ NSCs can switch to oligodendroglial progenitors by co‐expressing Olig2 with Nkx2.2 and Sox10. Thus, in most protocols, pluripotent stem cells are treated with SHH for the first 10 to 12 days in order to induce expression of Olig2. SHH can be replaced with purmorphamine^114^ or SAG.^115^ Dual inhibition of SMAD signalling with the combined use of the molecules SB431542 and LDN193189 has been shown to accelerate the production of Pax6+ NSCs and to upregulate Olig2, thereby generating the highest yield of Olig2+ progenitors.^115^, ^117^ This first part of differentiation, which includes the induction of neuroepithelium and then of Olig2‐expressing progenitors, generally takes place in adherent cultures.

Subsequently, continuous stimulation of SHH pathway is necessary to induce the formation of Olig2+, Nkx2.2+ pre‐OPCs. In contrast to the previous stage, transition from neuroepithelium to pre‐OPCs is best carried out as floating aggregates.^115^

Differentiation to Sox10+, PDGFR+ OPCs is a long process that may take up to 10 weeks. In order to boost the transition, most protocols introduce between the third and the fifth week a cocktail of factors known to drive oligodendrocyte differentiation or to promote oligodendrocyte survival, namely platelet‐derived growth factor (PDGF), neurotrophin 3 (NT3), triiodo‐l‐thyronine (T3), insulin‐like growth factor 1 (IGF‐1) and hepatocyte growth factor (HGF).^114^, ^115^, ^117^

Last steps of differentiation require the withdrawal of mitogenic factors and result in the production of O4+ immature oligodendrocyte and finally in the appearance of MBP+ ramified, mature oligodendrocytes.^115^

### 5.2 Generation of an iPSC‐based MSA model

MSA oligodendrocytes have been successfully generated by Djelloul and colleagues (Table 1).^43^ In their work, they generated iPSCs from skin fibroblasts of one MSA‐C patient, one MSA‐P patient, one familial PD patient and one healthy control, performing karyotype analysis in order to rule out possible chromosomal abnormalities. Subsequently, they differentiated them into functional oligodendrocytes using dual inhibition of SMAD signalling by small molecules LDN and SB and PDGF‐AA/IGF1/NT3/T3/HGF‐mediated terminal differentiation. Finally, they evaluated alpha‐synuclein expression by immunocytochemistry (ICC) and measured SNCA gene transcripts by real‐time PCR in the diverse lines at different stages of maturation. By day 60 in vitro, O4+, OLIG2+, PDGFRA+ oligodendrocyte progenitors showed the presence of SNCA transcripts and the expression of alpha‐synuclein, that was present also in neurons, while it was absent in astrocytes. Notably, the presence of alpha‐synuclein in oligodendrocytes decreased during maturation in all cell lines, including the diseased ones. Furthermore, they observed the relocation of alpha‐synuclein during oligodendrocyte differentiation from the processes to the perinuclear space. Interestingly, disease lines gave rise to an accelerated high yield of O4+ progenitors by day 70 in vitro.

To confirm these results, postnatal mouse forebrain primary cultures and mESC‐derived oligodendrocytic cultures were analysed for the presence of alpha‐synuclein and SNCA gene transcripts. Interestingly, in mouse brain primary cultures, alpha‐synuclein showed specific localization in B‐III‐Tubulin+ neurons and in O4+ oligodendrocytes. Analysis by real‐time PCR in both lines demonstrated the presence of SNCA transcripts in O4+ oligodendrocytes. A strong reduction in the expression of alpha‐synuclein was then observed in mature CNPASE‐ and MBP‐expressing oligodendrocytes, both in forebrain and in mESC‐derived cultures.

### 5.3 Relevance of the first iPSC‐based MSA model

This study suggests that oligodendrocytes might be able to produce alpha‐synuclein in vivo*,* at least during the first stages of maturation. However, the implications of these findings have not been completely understood yet. No significant differences in the expression of alpha‐synuclein were observed between healthy and diseased cell lines. This result might support the hypothesis that alpha‐synuclein found in MSA inclusions does not originate from oligodendrocytes, but on the contrary it is produced in neurons and subsequently taken up by oligodendrocytes. However, further research is needed to confirm this finding in a greater number of MSA‐derived iPSC lines. Neuronal‐oligodendroglial co‐cultures might be used to investigate possible mechanisms of synuclein uptake. Whether the accelerated production of immature oligodendrocytes in diseased cell could be ascribed to a greater oligodendrogenesis, probably triggered by cell injury, remains to be determined.

### 5.4 Promises and limitations of iPSC‐based models in MSA studies

Since their discovery, iPSCs have raised many hopes for the development of disease‐specific models that could replicate pathogenic events in vitro. Although in its infancy, MSA‐specific iPSC technology could be the key to solving some of the enigmas surrounding this disease. The advantages of this technique are that it is patient derived, and it allows to study neural and oligodendroglial maturation from their first stages. Therefore, it could be useful for elucidating mechanisms prior to alpha‐synuclein misfolding and accumulation, such as the origin of alpha‐synuclein in GCIs and the role of transcriptional upregulation of SNCA. Impairment of oligodendroglial precursors' maturation and myelination was shown to be involved in MSA pathogenesis.^33^, ^44^, ^69^ Thus, studying the various steps of oligodendrocytes generation and maturation through iPSC technology might help clarifying early events in MSA pathogenesis. Moreover, myelin lipids (sphingomyelin, sulfatide, and galactosylceramide) were found to be decreased in the affected white matter of MSA patients.^118^ These findings suggest that targeting lipid synthesis pathway at different steps of maturation might reproduce some of the neuropathologic features of MSA and possibly explain the extent of the contribution of myelin proteins dysregulation in disease pathogenesis.^118^ Another unresolved issue in MSA pathogenesis is whether neurodegeneration is a primary event or a consequence of oligodendroglial disfunction. Unravelling the pathways that lead to cell death is essential to find potential therapeutic targets. For example, recent in vitro studies highlighted the role of autophagy and defective intracellular clearance in alpha‐synuclein toxicity.^59^, ^61^, ^62^ Valera and colleagues targeted a specific regulator of autophagy, microRNA‐101, in order to create a model of MSA and to identify potential therapeutic agents.^61^ iPSCs can be differentiated into many cell types and thus it could be possible to study degenerative pathways both in affected neurons and in oligodendrocytes. Moreover, patient‐derived iPSCs could generate not only simple oligodendroglial or neuronal cultures, but also mixed oligodendroglial‐neuronal cultures, which could then be searched for differential expression of alpha‐synuclein, apoptotic markers and other relevant proteins. Mixed oligodendroglial and neuronal cultures could also represent a useful tool to study trophic interaction between neurons and oligodendrocytes. A reduction in GDNF was observed in transgenic mouse models of MSA, and it is believed to play a role in neurodegeneration.^56^ Co‐cultures could expand our knowledge about the role of GDNF and other trophic factors in the neuropathological alterations found in MSA. Furthermore, both direct intercellular transmission mechanisms^47^, ^48^, ^49^ and exosomes^73^, ^74^, ^75^ were shown to be involved in alpha‐synuclein propagation. These interactions could be better analysed in co‐cultures. Another exciting possibility is the creation of *organoids*, iPSC‐based tridimensional cellular cultures, which reproduce human brain tissues with their different cell types and complex cellular interactions.^119^ Although these techniques have not been used for studying MSA so far, significant advancements have been made in other fields.

On the other hand, studying a disease such MSA with iPSCs poses many challenges. The absence of a “smoking gun” gene defect means it is extremely difficult to model MSA with stem cells. Likewise, the lack of a precise knowledge about the role of environmental influences in the pathogenesis challenges the validity of in vitro results. Hence, many different diseased cell lines and controls would be necessary to state that a finding is not an artefact, making research of this kind long and expensive.

Having made these points, iPSCs still remain the best technique to study MSA at a preclinical level so far. Both animal models and primary cultures require some kind of manipulation to overexpress alpha‐synuclein, despite it is not known whether such a mechanism is responsible or not for MSA pathogenesis. As genetic research about MSA is making important progresses, it is possible that in a near future we will be able to identify mutations or polymorphisms associated with the disease.

## 6 CONCLUSIONS AND FUTURE PERSPECTIVES

Despite the many studies that have been conducted, MSA pathogenesis remains elusive. Surely, the paucity of reliable disease models represents a significant obstacle for researchers. Different groups attempted to reproduce the features of the disease by genetically reprogramming primary glial lines.^61^, ^69^, ^105^, ^106^ These models were useful to investigate the effects of the overexpression of alpha‐synuclein and p25alpha on oligodendrocytes' maturation and survival. It was observed that the excessive production of alpha‐synuclein at an oligodendroglial level causes the formation of fibrillary alpha‐synuclein aggregates and favours cell death.^106^ In another study, the concomitant expression of p25alpha was shown to induce alpha‐synuclein aggregation and the subsequent activation of the apoptotic cascade.^105^ Furthermore, it was seen that overexpression of alpha‐synuclein in OPCs impaired their normal maturation and myelination, possibly through a downregulation of myelin‐associated genes.^69^ In addition to that, the presence of a dysregulation of autophagic pathways and its contribution to the intracellular deposition of alpha‐synuclein was analysed.^61^ However, these models have several flaws, as they are not based on patient‐derived cultures and thus they do not share the same genetic background as patients. Moreover, it is not known whether alpha‐synuclein is primarily overexpressed in oligodendrocytes or if MSA neuropathological features originate from the interaction between neurons and oligodendrocytes. Therefore, models based on primary cell cultures are of limited use in drug testing.

Conversely, the advent of iPSC technology opens up thrilling possibilities for the study of neurodegenerative diseases such as MSA. iPSC‐based models are human relevant, and they retain the same genetic inheritance as the patient. Thus, they raise hopes on the possibility of testing drugs in a safe and reliable manner, and developing treatments based on autologous stem cell transplantation. Furthermore, the use of stem cells allows researchers to observe neurons and glial cells during their differentiation and maturation, making it possible to identify early events that could trigger late neurodegeneration. Djelloul and colleagues were the first to generate oligodendrocytes from iPSCs of patients with MSA. Their work shows that alpha‐synuclein is produced in oligodendrocytes progenitors and registers a significant decrease during maturation, but no differences were observed between healthy and MSA cell lines.^43^ However, it was noted that disease lines generated a higher yield of O4+ progenitors at an accelerated rate. Although these results suggest the hypothesis that GCIs' alpha‐synuclein might not originate from oligodendrocytes, further models are needed to confirm these findings.

iPSC technology also poses a variety of challenges. The absence of known genetic or environmental culprits makes it difficult to determine whether MSA is one disease or many. As a consequence, the validity and significance of in vitro results obtained from patients' iPSCs needs to be confirmed with a great number of observations.

In conclusion, although we are far from the understanding of disease mechanisms, future research may take advantage from the uncountable opportunities offered by IPSCs. For example, co‐cultures of neurons and oligodendrocytes may shed light on the cellular origin of alpha‐synuclein. Furthermore, iPSC‐based tridimensional cellular cultures, or *organoids,* may provide an excellent insight into MSA pathogenesis, as they reproduce complex cellular interaction in a near‐physiological environment.^119^ In addition to that, MSA‐derived iPSC cultures may allow scientists to focus on molecular changes that occur in patients prior to neurodegeneration or symptoms onset. The identification of markers of subclinical disease may be the first step towards early diagnosis and effective pharmacological interventions.
